# Physical Fitness in Children with Type 1 Diabetes Measured with Six-Minute Walk Test

**DOI:** 10.1155/2013/190454

**Published:** 2013-06-27

**Authors:** Vladimir Jegdic, Zeljko Roncevic, Veselin Skrabic

**Affiliations:** ^1^Department of Pediatrics, University Hospital, 88000 Mostar, Bosnia and Herzegovina; ^2^Department of Pediatrics, University Hospital, 21000 Split, Croatia

## Abstract

*Aim/Hypothesis*. To examine whether children with DMT1 are less physically fit than healthy children and to assess whether an elevated level of HbA1c was associated with decreased physical fitness among children with diabetes. *Methods*. The study was conducted using case-control methodology. The cases were 100 children with T1DM, 7–17,9 years. Study subjects underwent a 6MWT, where distance measured, heart rate, and oxygen saturation was recorded. *Results*. Results of the 6MWT for children with T1DM and controls were 601.3 ± 86.1 meters versus 672.1 ± 60.6 meters, respectively (*P* < 0.001). The cases were divided into two subgroups, one with HbA1c levels >8% and one with HbA1c <8%. Results for both groups were inferior to the controls (*P* < 0.001). The posttest pulse rate in all subjects was higher than the pretest pulse rate (*P* < 0.001). Pulse oxygen levels were lower than controls at the pretest measurement (*P* < 0.001), and for both cases and controls, pulse oxygen levels decreased after test (*P* = 0.004). However, the change in oxygen saturation did not differ between the groups (*P* = 0.332). *Conclusions*. Children with T1D are less fit than matched controls. The level of HbA1c did not affect the physical fitness of children with T1D.

## 1. Introduction

Diabetes mellitus type 1 is a multifactorial autoimmune disease characterized by complete destruction of pancreatic beta cells and loss of insulin production, caused by multiple genetic and environmental influences [1].

Young people with type 1 diabetes (T1D) have been found to have decreased aerobic capacity and lower cardiorespiratory fitness levels compared to nondiabetic control subjects [2, 3].

Serum concentration of HbA1c is the “gold standard” for the assessment of both therapeutic efficacy and the risk of development of diabetic microvascular and macrovascular complications [4].

Various factors are associated with glycemic control including age, sex, diabetes duration, and management (frequency of blood glucose monitoring, insulin regimen, and dose adjustments), as well as those indirectly connected to diabetes care (family history, dietary, cultural habits, etc.) [5].

Physical activity also plays an important role of glycemic control. Nondiabetic individuals have a reduction in insulin secretion and an increase in glucose counterregulatory hormones that facilitate an increase in liver glucose production that matches skeletal muscle glucose uptake during exercise, and consequently glucose levels during physical activity remain stable [6, 7].

In patients with T1D the pancreas does not regulate insulin levels in response to exercise, and there may be impaired glucose counterregulation, making normal fuel regulation nearly impossible [8]. In T1D patients with poor glycaemic control, there is insufficient amount of insulin, and counterregulatory hormones induced by physical activity will cause a further increase in blood glucose levels. In contrast, increased amounts of insulin present in the circulation will reduce or even prevent the mobilization of glucose which can result with hypoglycemia [9]. Regular physical activity has a particularly positive effect by improving insulin sensitivity and allows for a better use of the synthesis of glycogen and fat as energy sources [7, 10].

The six-minute walk test (6MWT) is a quick, simple and inexpensive method of determining physical fitness. It is also an important clinical test used to determine the quality of life [11]. It measures the distance achieved during a smooth walk with a constant effort for six minutes on a flat, hard surface. This is a simple method for estimating the physical stamina during submaximal effort. It is routinely used to assess the condition of people with cardiovascular and pulmonary diseases [12]. Initially the test was twelve minutes long and was used to test the healthy population. Cooper used it in the study of physical fitness of American pilots [13]. The test has been used in evaluation of patients with chronic obstructive pulmonary disease and the American Thoracic Society has developed guidelines for its use [14, 15]. Additionally the 6MWT has been used for monitoring the course of disease and response to therapy in patients with rheumatism, pulmonary, cardiovascular, and endocrine diseases [14–25]. Factors that have been shown to affect the test results include age, gender, leg length, body weight, various diseases, and the motivation of the subjects undergoing testing [15]. In children the 6MWT is being increasingly used [26–28].

## 2. Objective

The objectives of this research are

1. investigate whether children with T1D are less physically fit than healthy children;
2. establishing the influence of HbA1c level on physical fitness.

## 3. Subjects and Methods

### 3.1. Patients

We examined all children (186 children) aged 7–18 from Split—Croatia and Mostar—Bosnia and Herzegovina who have type 1 diabetes.

After examining medical history from the study children with cardiorespiratory disease and anemia were excluded. Also, patients with blood glucose levels were excluded (below 4, 0 and above 14 mmol/L), just prior to exercise testing or who tested positive for ketone.

The study group consisted of 100 children with T1D aged 7–18 years from the Mostar—Bosnia and Hercegovina and Split—Croatia, without clinical cardiopulmonary disease or anemia.

The control group consisted of the same number of healthy individuals from primary and secondary school, of equal age and sex, with a similar height (not greater than 2 cm), weight (not greater than 2 kg), length of the lower extremities, BMI, and who were not acutely ill a month before the test.  Table 1 presents the demographic and anthropometric measures for the cases and controls.

At a predetermined arrangement with the principal and class, children were provided with informational flyers that were submitted to parents for review and eventual approval. Signed approval was given by parents and the participant. The study was approved by the Ethics Committees of the University Hospital Mostar and the University Hospital Split. The diagnosis of T1D was made based on the criteria of the American Diabetes Association [29].

### 3.2. Anthropometric and Other Measurements

The participants weight (kg) and height (cm), body mass index (BMI—weight in kg divided by height in meters squared), heart rate (beats per minute), blood pressure (mm Hg), the lower segment of the body from the upper edge of the symphysis to the floor in upright position (cm), and blood oxygen saturation SpO_2_ (%) (CMS-50E fingertip Pulse Oximeter OLED) were determined in order to match participants and compare their performance.

In a group of type 1 diabetic patients HbA1c was measured within ±10 days of 6MWT by spectrophotometery (DCA 2000+, Siemens, Germany). Capillary blood glucose (CBG) level measured by a glucose meter was performed before and after the 6MWT. All patients had breakfast and an insulin injection administered 2 h to 2,5 h earlier.

### 3.3. 6MWT

The test consisted of the six-minute walking on a flat, hard surface 20 meters long (20 meters forward and 20 meters back). Participants used the measuring wheel (Nedo GmbH & Co. KG, Dornstetten, Germany) which was held by hand. The measuring wheel has different handle lengths (240, 370, and 560 mm) that were changed depending on the height of participant. Each participant chose the handle of appropriate length. Participant had not engaged in serious physical activity for at least two hours before the test.

The test was performed in a separate room, and a dial on the wheel was covered during the performance test to rule out the possibility of competition between participants.

On the floor a distance of 20 meters was measured and marked by cones. Each participant was given the opportunity to experience the wheel and select the optimal handle size. Then he was given instructions for the test which read: “The purpose of this test is to walk as fast as you can in six minutes and cross as many meters you can. You will walk around the set cones. You are allowed to slow down and stop, if necessary, and lean on the wall and when you can, continue. It is forbidden to run. Again, the purpose is to cross as many meters as you can. If you're ready, go!” Time was measured by a stopwatch, and every minute the respondents were informed by standard phrases: after the first minute, “Well done, you have five minutes to the end,” after another minute, “Keep it up, you got four more minutes,” after three minutes, “Well done, you're half-way,” after four minutes, “Keep it up, you got two more minutes,” and after five minutes, “Just keep going, you have another minute.” No other words of encouragement as well as body language were used to avoid the possibility of encouraging the participant to speed up or slow down. The examiner at all times stood in the middle of the walking track to control the accuracy of the test. After six minutes the participant was asked to stop. Immediately after that participant was placed in a sitting position where blood oxygen saturation and heart rate were measured. Then we noted how many meters the participant had crossed in six minutes.

### 3.4. Statistical Analysis

Statistical analysis included the Kolmogorov-Smirnov test for symmetry of distribution of continuous variables. For a description of their assembly and disintegration the arithmetic mean and standard deviation was used. Comparison of two normally distributed independent variables was performed using Student's *t*-test. Comparison of continuous variables measured at multiple time points was made by the test of repeated measurements. Unlike the distribution of nominal variables an ordinal *χ* ^2^ test was used. The possibility of a type I error was set at *α* < 0.05 and differences between groups were accepted as statistically significant at *P* < 0.05.

For statistical analysis we used SPSS for Windows (version 13.0, SPSS Inc. Chicago, IL, USA) and Microsoft Excel (version 11, Microsoft Corporation, Redmond, WA, USA).

## 4. Results

The study and control groups included 49 girls (49.0%) and 51 boys (51.0%), and there were no significant differences in sex ratio (*χ* ^2^ test = 0.040, df = 1, *P* = 0.841). The average age was 13.0 ± 2.9 years (mean ± SD), range 7.0 to 17.9 years.

Test and control groups did not differ significantly in average height (Student *t*-test = 0.073, *P* = 0.942), in average body weight (Student *t*-test = 0.046, *P* = 0.964), in the average length of the lower extremities (Student *t*-test = 0.045, *P* = 0.964), and in mean body mass index (Student's *t*-test = 0.043, *P* = 0.955) (Table 1).

Combining both cases and controls, the average value of the 6MWT was 636.7 ± 82.3 meters, range (360.3–808.0) meters.

The cases had significantly shorter distance measured in meters compared to the control group (Student *t*-test = 6.718, *P* < 0.001) (Table 2).

According to the values of HbA1c test group was divided into two subgroups. The first group consisted of children with the values of HbA1c <7.9% (*n* = 40). In the second group children with the values of HbA1c ≥8.0% (*n* = 60) were included. Both subgroups were compared with their pairs in the control group.

Children with lower levels of HbA1c of 8.0% exceeded by significantly shorter distance the corresponding control group (Student's *t*-test = 4.109, *P* < 0.001) (Table 3).

Children with levels of HbA1c ≥8.0% walked a significantly shorter distance than the corresponding control group (Student *t*-test = 5.279, *P* < 0.001) (Table 4).

Observing the entire sample, cases, and control groups pretest heart rate was significantly slower (88.3 ± 14.7), compared to the heart rate after the test (131.2 ± 20.7) (*F*(1.198) = 931.573; *P* < 0.001).

**Figure 1:**
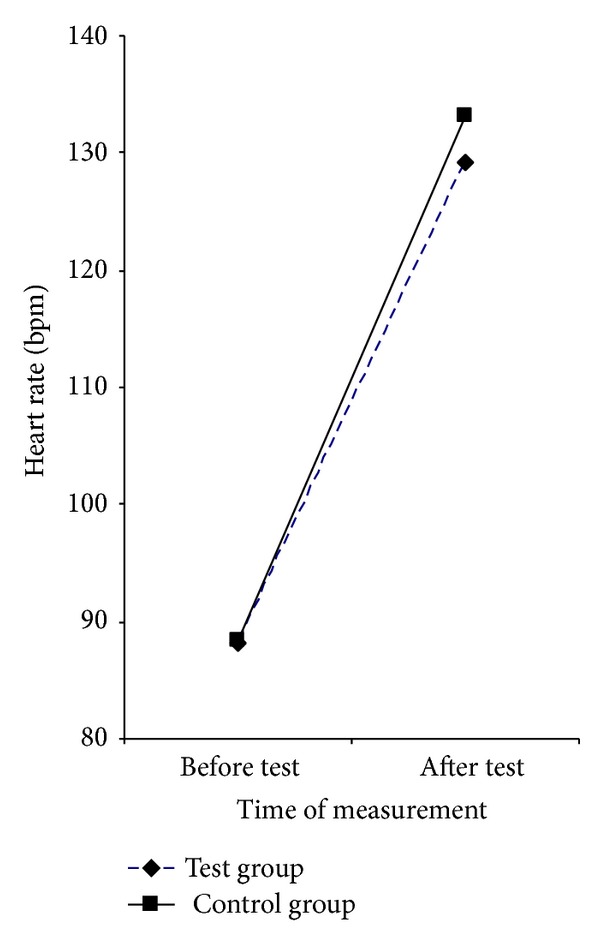
Presentation of the groups studied in relation to the value of the heart rate before and after the test.

There were no significant differences in heart rate before and after the test between the two groups (*F*(1.198) = 1.733, *P* = 0.190) (Figure 1).

Observing the entire sample, cases, and control group, oxygen saturation (%) before the test was significantly higher (98.8 ± 1.0) compared to the oxygen saturation (%) after the test (98.6 ± 1.0) (*F*(1.198) = 8.529, *P* = 0.004).

A downward trend in the oxygen saturation between the two groups, before and after the test, showed no statistically significant difference (*F*(1.198) = 0.948, *P* = 0.332).

**Figure 2:**
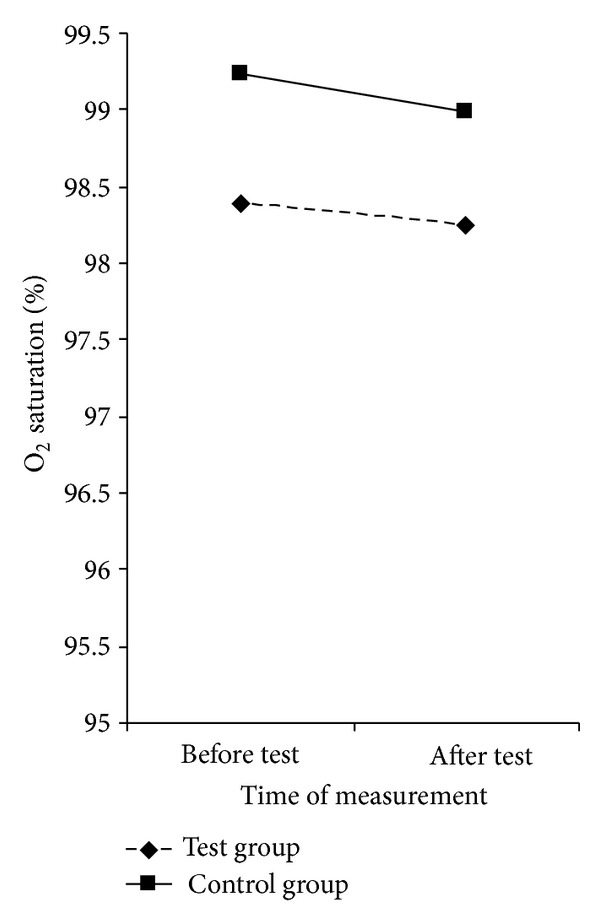
Presentation of the groups studied compared to the value of oxygen saturation before and after the test.

Taking into consideration the overall value of oxygen saturation before and after the test, the test group had significantly lower values of saturation (98.3 ± 1.1) compared to the control group (99.1 ± 1.1) (*F*(1.198) = 51.238, *P* < 0.001) (Figure 2).

## 5. Discussion

In our study, we compared the physical fitness of children with T1D with controls. The results of our study indicate that children with T1D are less physically fit than a matched set of healthy control children (Table 1). 

Physical activity has a positive effect on health [30]. The ability of trained muscle to take and oxidize free fatty acid reduces blood lipid levels. The positive effect on blood pressure significantly reduces the overall risk of cardiovascular disease [31]. Maintaining fitness and ideal body weight promotes self-esteem and self-satisfaction [32]. All these effects are particularly important in patients with diabetes who have chronic hyperglycemia as they are exposed to additional risks. Regular physical activity improves insulin sensitivity and reduces the daily need for exogenous insulin. In children with T1D the frequency of regular physical activity was associated with lower HbA1c without increasing the risk of severe hypoglycemia [33].

Despite the positive effect that physical activity has on blood glucose, controlled studies have not confirmed a long-term improvement in metabolic control in patients with T1D. Physical activity may be just one element in the complex therapy of diseases. Another possible explanation is that patients avert the risk of hypoglycemia before physical activity by underdosing insulin or by injecting carbohydrate, and this offsets the benefits of exercise [34].

The 6MWT is a reliable test for the assessment of physical fitness [11]. In many countries all over the world the standards of normal values for different age groups have been established [26–28, 35]. The test can be used to monitor the progress of disease and response to therapy in patients with various diseases. Dos Santos Alves et al. used the 6MWT for monitoring lung capacity in patients with idiopathic scoliosis [16]. Otto Lelieveld used the 6MWT in children with juvenile idiopathic arthritis [17]. However, the most common use of 6MWT is in pulmonary and cardiovascular diseases [18–23]. Novak et al. applied 6MWT in diabetics who have neuropathy as a complication of their primary disease and showed that patients with severe leg pain have more difficulty in walking than patients with mild pain or no pain which significantly affects their quality of life [24] 6MWT represents a practical and reliable assessment tool for exercise performance in overweight and obese children and adolescents [36, 37].

To the best of our knowledge in the literature, we have not found studies that used the 6MWT in children with T1D. We found results documented from one pilot study. Physical condition of seven type 1 diabetic girls aged 8–10 years was given exercise scheme activities and examined with a 6MWT before and three months after [25].

Thanks to the precise regulation in nondiabetic individuals glucose levels during physical activity remain stable [6, 7]. In T1D the pancreas does not regulate insulin levels in response to exercise, and there may be impaired glucose contrarregulation. In poor controlled diabetic patients, there is an insufficient amount of insulin, and contraregulatory hormones in physical activity cause a further rise in blood glucose levels. In contrast, the increased amount of insulin present in the circulation will reduce or even prevent the mobilization of glucose which can result in hypoglycemia; it may explain the weaker results of physical fitness of the group with T1D [38]. Although we hypothesized that that subjects with well-controlled diabetes would perform better on the 6MWT than those with poorly controlled diabetes [39], our results did not confirm this (Tables 2 and 3). The study group was divided into two groups: well and poorly controlled. A HbA1c of 8.0% was taken as the dividing boundary. Then these groups were compared with their controls. It is known that HbA1c reflects levels of glycemia over the preceding 4–12 weeks [7]. The reason why is this value taken as the limit comes in the following facts. From the clinical side of view, we can say that the value of HbA1c of 7.9% and less is acceptable especially if it is known that the majority of respondents were in puberty, when many adolescents experience a deterioration in metabolic control. One of the reasons for this is the greater insulin resistance at puberty [40].

The difference in heart rate frequency was statistically significant when comparing values before and after the test, which means that all respondents gave their maximum during the performance test (Figure 1). It is also very important to note that there was no difference in heart rate frequency between the groups suggesting that both groups were equally motivated and goal-directed in the test. The better result of physical fitness in the control group is not a result of greater effort than the test group. Given that the cases were free of vascular complications of diabetes, the difference in oxygen saturation between the groups was surprising (Figure 2). This could be explained by possible functional and structural abnormalities in the peripheral blood vessels caused by diabetes and poor cardiorespiratory fitness of these patients, although the trend of oxygen saturation in both groups remained the same. The question remains whether the reduced performance of children with T1D is associated with lower levels of physical activity or is it a result of their illness [3, 41]. Children with T1D have been found to have decreased aerobic capacity measured by VO2 max and also to have lower heart rate at exercise exhaustion compared to nondiabetic control subjects. The authors postulated that individuals with T1D had decreased lung ventilation associated with decreased maximal O_2_ consumption and exercising capacity [2]. Children with T1D aged 5–14 years had reduced cardiorespiratory fitness levels compared to nondiabetic control children [3]. Recent research in patients with type 2 diabetes reports less blood flow to the periphery in physical activity [42]. As the oxygen saturation is measured at the fingertip, this may explain our result.

## 6. Conclusions

We found that children with T1D are less physically fit than matched healthy controls as measured by the 6MWT.

We also found that the level of HbA1c did not affect the physical fitness of children with T1D.

Future research is needed to confirm these results and should investigate whether the reduced physical fitness in children with T1D is attributable to physiological changes resulting from the diabetes pathology itself.

## Figures and Tables

**Table 1:** Presentation of the groups studied in relation to body height, weight, length of the lower extremities, and BMI.

Variables	Mean ± standard deviation		P*
	Test group	Control group	
Number of participants	100	100	
Height (cm)	159.9 ± 14.3	160.1 ± 13.9	0.942
Weight (kg)	51.1 ± 15.0	50.98 ± 14.7	0.964
Lower extremities length (cm)	78.9 ± 8.1	78.8 ± 7.5	0.964
BMI (kg/m^2^)	19.4 ± 3.5	19.5 ± 3.3	0.955

*Student *t*-test.

**Table 2:** Presentation of the groups studied in relation to the mileage meter.

Variables	Mean ± standard deviation		P*
	Test group	Control group	
Number of participants	100	100	
Crossed distance	601.3 ± 86.1 m	672.1 ± 60.6 m	<0.001

*Student *t*-test = 6.718; *P* < 0.001.

**Table 3:** Presentation of the groups studied with HbA1c levels lower than 8.0% and their controls in relation to the mileage meter.

Variables	Mean ± standard deviation		P*
	Test group	Control group	
Number of participants	40	40	
Crossed distance	599.8 ± 86.7 m	669.2 ± 62.3 m	<0.001

*Student *t*-test = 4.109; *P* < 0.001.

**Table 4:** Presentation of the groups studied with the level of HbA1c ≥8.0% and their controls in relation to the mileage meter.

Variables	Mean ± standard deviation		P*
	Test group	Control group	
Number of participants	60	60	
Crossed distance	602.4 ± 86.4 m	674.1 ± 59.9 m	<0.001

*Student *t*-test = 5.279; *P* < 0.001.
